# Uniaxial Tension Test of Slender Reinforced Early Age Concrete Members

**DOI:** 10.3390/ma4081345

**Published:** 2011-08-02

**Authors:** Yoichi Mimura, Isamu Yoshitake, Wenbo Zhang

**Affiliations:** 1 Department of Civil and Environmental Engineering, Kure National College of Technology, Agaminami 2-2-11, Kure, Hiroshima 737-8506, Japan; 2 Department of Civil and Environmental Engineering, Yamaguchi University, 2-16-1 Tokiwadai, Ube, Yamaguchi 755-8611, Japan; E-Mails: yositake@yamaguchi-u.ac.jp (I.Y.); n005we@yamaguchi-u.ac.jp (W.Z.)

**Keywords:** cracking, tensile stress, bonding

## Abstract

The present study aims to obtain the tensile properties of early age concrete based on a uniaxial tension test employing RC slender members. First, the paper shows that concrete strain is equal to the strain of rebar at the mid-span of the RC member. The tensile Young’s modulus and the strain capacity of early age concrete are estimated using strain measurements. The experiment indicated that the tensile Young’s modulus at an early age is higher than the compressive modulus. This observation was similar to one found in a previous investigation which used a direct tension test of early age concrete. Moreover, the paper describes how an empirical equation for mature concrete can be applied to the relation between uniaxial tensile strength and splitting tensile strength even in early age concrete. Based on a uniaxial tension test, the paper proposes an empirical equation for the relationship between standard bond stresses and relative slip.

## 1. Introduction

The mechanical properties of concrete vary according to the degree of hydration as the strength develops with the age of the concrete. On the other hand, concrete volume always changes due to hydration heat during early age active reactions. Early age cracks often occur when the tensile stress due to the volume changes arising from thermal contraction and shrinkage is higher than the tensile strength at that age. To predict and prevent such cracking, designers and conductors often use a thermal stress simulation [1]. Such simulations require the mechanical properties of concrete to predict restraint stresses from the volume changes. Early age tensile characteristics are especially important for such predictions.

Many investigations dealing with early age tensile properties have been conducted for predicting cracking. ACI Committee 231 presented a report of early age cracking based on the latest studies [2]. The report describes causes and measurements of cracking and presents methods to mitigate cracks. Although such reports have been frequently published, prevention of early age cracking is still a major concern of concrete engineers. For example, Bergstrom and Byfors reported properties of early age concrete based on numerous investigations [3]. Their report includes thermal properties of concrete as well as strengths and deformations. They pointed out that, in addition to time, early age concrete should be defined by its hydration degree because temperature strongly affects the performance of concrete. Houghton presented tensile strain capacity based on flexural tests using simple concrete beams [4]. His report emphasizes that a rapid testing method is required for estimating early age tensile strain capacities. Jin and Li conducted a direct tension test which bonded concrete and loading jigs via epoxy adhesive in order to obtain the tensile properties of early age concrete [5]. Young’s moduli in their tests were relatively small. In particular, it was remarkable that tensile Young’s moduli at an early age were smaller than compressive moduli. These observations differ from several previous reports [6,7,8]. This difference may be caused by the difference in testing methods. Swaddiwudhipong et al. also investigated tensile Young’s moduli of early age concrete by using a direct tension test [9]. Their test included ground granulated blast furnace slag and pulverized fuel ash in concrete. They reported that the tensile Young’s moduli of all the concrete samples were well-correlated with tensile strength. Kim *et al*. investigated fracture characteristics of early age concrete by using wedge-splitting specimens [10]. They proposed bilinear softening curves for load-crack relations width based on the experiments and FE simulations.

Chapman and Shah conducted pull-out tests of rebar from early age concrete to quantify bond strength [11]. They reported that the bond strength of deformed bars was significantly affected by age. Hughes and Videla experimentally investigated early age bond strength by employing various deformed bars and other methods [12]. Their test was to pull two rebars from both ends of a concrete bar, so the concrete was constantly in a tensile stress field. Sule and Breugel investigated the cracking behavior of an RC member of high strength concrete using the temperature stress testing machine (TSTM) [13]. As a fundamental test, pull-out tests were also conducted to obtain bond development of rebar at a very early age, *i.e*., 8, 24, 31 hours. The bond strength was evaluated as bond stress at slips of 0.1 mm and 1 mm. The values calculated from the predicting formula were approximately 30% lower than the early age experimental values. In addition, Sule and Breugel presented crack formation of an early age RC member [14]. They found a micro crack in the vicinity of the rebar, which was due to restrained early age volume changes. It should be noted that the object of their study was only high strength concrete, which is characterized by significant autogenous shrinkage.

As summarized above, a number of investigations dealing with mechanical properties of early age concrete have been conducted. However, little research on the cracking behaviors of concrete with a long bond length of rebar has been conducted. By employing a uniaxial tension test with RC members, early age bond behavior may be appropriately obtained. In addition, the test method using bond properties of rebar in concrete may yield some early age mechanical properties, e.g., tensile strength and tensile strain capacity. This paper describes the tensile properties of normal strength early age concrete based on such a laboratory test.

## 2. Experimental Program

### 2.1. Materials and Mix Proportion of Concrete

The present study uses the materials shown in Table 1 for concrete. The cement employed is blast furnace slag cement [15], which is often used for construction of infrastructure in Japan. The fine aggregate and gravel are also normal materials in western Japan. Table 2 gives the mix proportion of the concrete. To investigate the properties of normal concrete, this study used the concrete with a mix proportion often used in a ready-mixed concrete factory.

**Figure 1:**
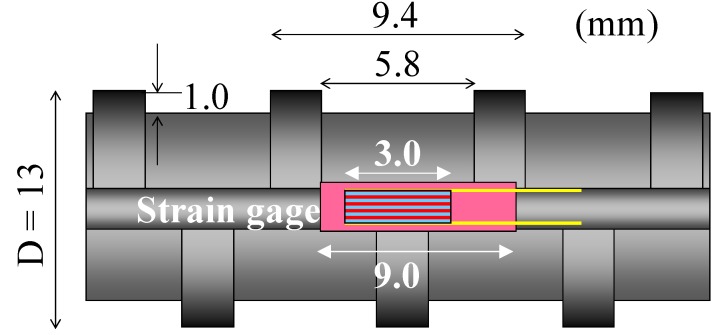
Deformed steel bar.

Deformed steel bars used for reinforced concrete specimens are described in Figure 1. The reinforcing steel showed a yield strength of 350 GPa with an elastic modulus of 190 GPa (measured) and a tensile strength of 483 GPa. The minimum yield strength of the reinforcing bar (SD295A) was 295 GPa [16].

**Table 1:** Materials for concrete.

***C*: blast furnace slag cement (B) JIS R 5211**	
density	3.04 g/cm^3^
blaine fineness	3770 cm^2^/g
setting time start-end	3 h. 39 m.–4 h. 57 m.
comp. strength at 3day	21.5 MPa
at 7day	37.7 MPa
at 28day	63.2 MPa
***S*: fine aggregate (sea sand)**	
density	2.56 g/cm^3^
maximum size	5 mm
absorption	1.3 %
***G*: coarse aggregate (crushed andesite rock)**	
density	2.73 g/cm^3^
max. / min. size	20/5 mm
absorption	0.6%
***Ad*: chemical admixture (AE water reducing agent)**	
standard amount for use	1.0% vs cement weight
density	1.08 g/cm^3^

**Table 2:** Mix proportion of concrete.

**Water cement ratio (*W/C*)**	0.57 (57%)
**Water *W***	165 kg/m^3^
**Blast furnace slag cement *C***	290 kg/m^3^
**Fine aggregate *S***	812 kg/m^3^
**Coarse aggregate *G***	1030 kg/m^3^
**Admixture *Ad***	2.9 kg/m^3^
**Air**	4.5%

### 2.2. Mechanical Properties of Concrete

The uniaxial tension tests were conducted to obtain tensile properties of concrete at early age. For a comparison, compressive strengths, splitting tensile strengths and compressive Young's moduli were also tested. Table 3 summarizes the test and the number of specimens.

**Table 3:** Tests and number of specimens.

**Age (days)**	0.5	1	1.5	2	3	7	28
Compressive strength test*	3	3	3	3	3	3	3
Splitting tensile test**	3	3	3	3	3	3	3
Uniaxial tension test***	1	1	1	1	1	1	1

*Compressive strength test: Compressive strengths (*f’_c_*) and compressive Young’s moduli (*E_Cc_*); **Splitting tensile test: Splitting tensile strengths (*f_t_*_2_); ***Uniaxial tention test: Tensile Young’s moduli (*E_Ct_*), cracking stresses (*f_t_*_1_), Tensile strain capacities and Bond properties.

**Figure 2:**
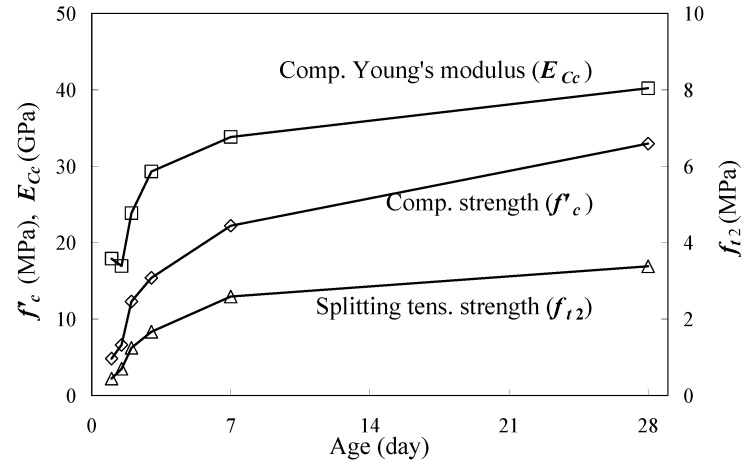
Compressive strength, splitting tensile strength and compressive Young’s modulus.

The fundamental properties of the concrete, shown in Figure 2 are compressive strengths (*f’_c_*), splitting tensile strengths (*f_t_*_2_) and compressive Young’s moduli (*E_Cc_*) developing with age. This study used 3 cylindrical specimens (100 × 200 mm; diameter × height) for each test. Herein, the tests were conducted referring to JIS-A-1108 [17], JIS-A-1149 [18] and JIS-A-1113 [19] respectively. The test results showed mechanical properties of the concrete significantly developed before the age of 7 days, so the study aimed to investigate tensile behaviors in that age range. The test ages were 0.5, 1, 1.5, 2, 3, 7 and 28 days. All concrete specimens had been cured under a wet condition of 20 °C.

### 2.3. RC Specimen

**Figure 3:**
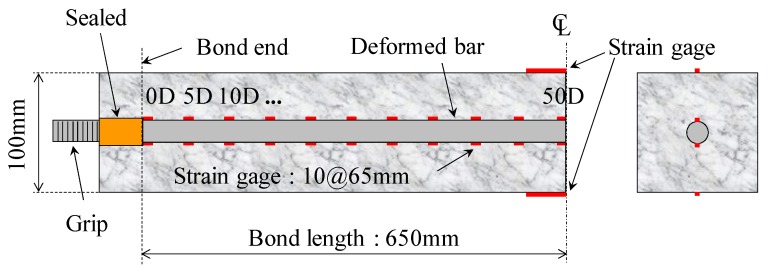
A specimen for the uniaxial tension test.

Figure 3 shows a specimen (100 × 100 × 1560 mm) with a reinforcing bar embedded in the center section. The experiment used electric resistance wire strain gages 3 mm long for rebar and 20 mm long for concrete, respectively. To measure strain distribution of the rebar, the gages were attached on each lateral rib of the rebar using an adhesive. The short gages were attached 65 mm (5D) from the mid-span. The long gages were bonded to the surface of the concrete at mid-span in order to be compared with the strain of the embedding rebar.

### 2.4. Uniaxial Tension Test

**Figure 4:**
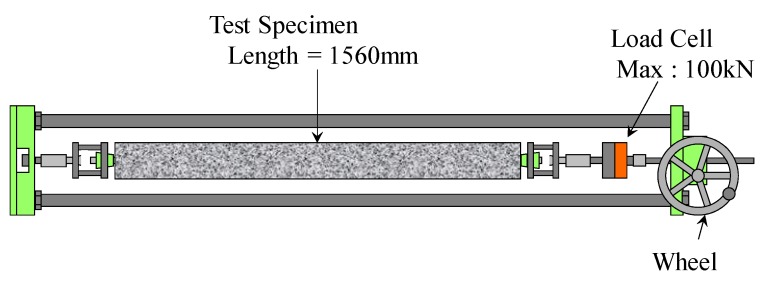
Set up of the uniaxial tension test.

Both ends of the rebar were attached to a loading jig, and each of the end conditions was rotatable. Figure 4 presents an apparatus providing a tensile force to the specimen. The specimen can be easily loaded to the apparatus by human-power, and the force was measured by using a load cell that has a capacity of 100 kN. By steering the wheel of the apparatus, the tensile force was increased with a rate of loading of 0.1 kN/s approximately. The investigation measured the force and the strains with an interval of 1.0 kN approximately. The maximum force in the test was 35.1 kN, so the test reported in the paper does not include the behavior of yielded rebar. It is noted that all concrete specimens had a first crack near mid-span during the loading. Herein, the mid-span means the longitudinal center of the RC slender specimens.

## 3. Cracking Behavior of Early Age Concrete

### 3.1. Evaluation Method for Stress of Concrete

**Figure 5:**
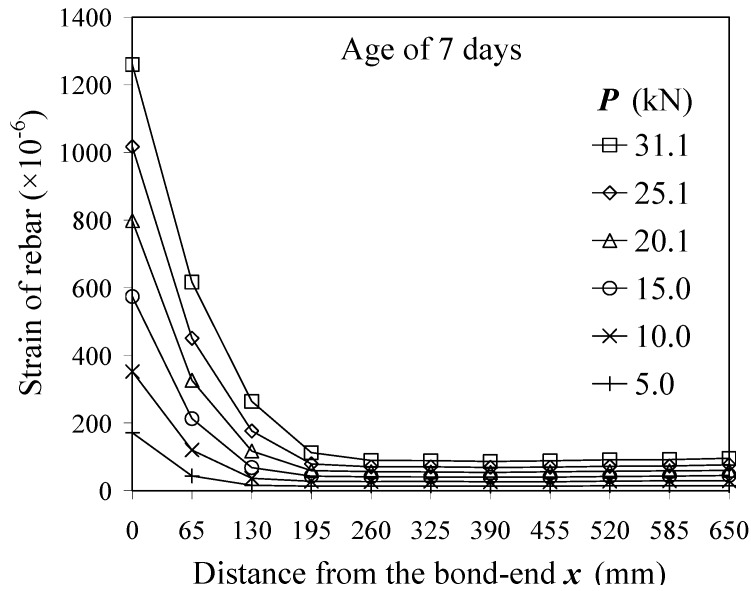
Strain distributions of the rebar embedded in the concrete (at 7 days old).

**Figure 6:**
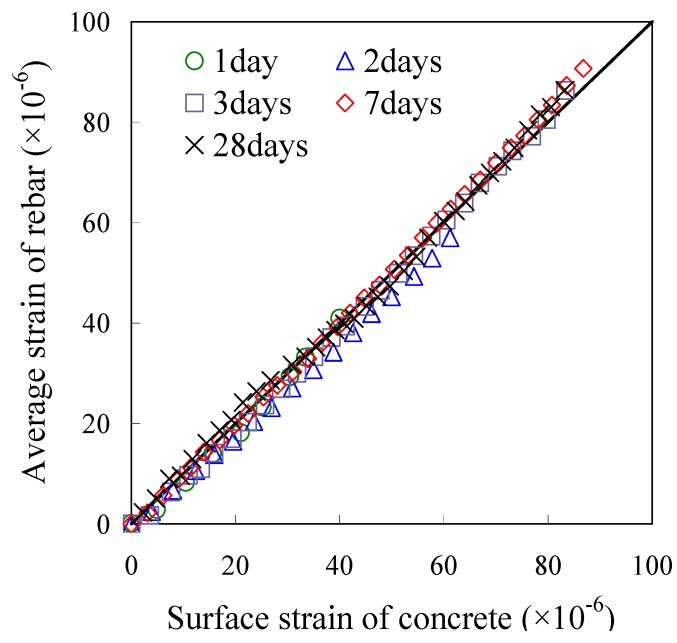
Surface strain of concrete *vs.* strain of rebar (390 mm range from the mid-span).

**Figure 7:**
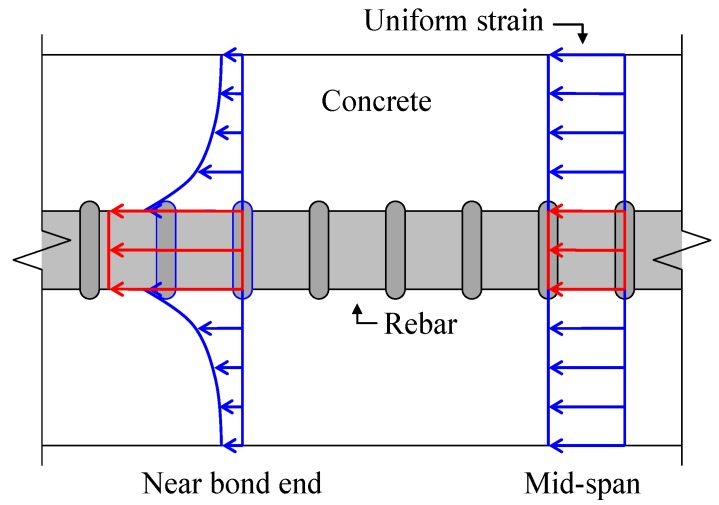
Strain distributions in cross section of specimen.

Figure 5 presents a typical strain distribution of the rebar embedded in the concrete. The strains within 390 mm of the mid-span were constant at each tensile force. Figure 6 shows a comparison of the constant strains and the surface strain of concrete. It is obvious that strains of rebar near the mid-span were perfectly equal to the surface strain. This result shows that the concrete strain in the cross section was uniform as shown in Figure 7, and equal stress could act upon the concrete section near the mid-span. This method may reduce bending moments due to a local deformation of concrete.

Equation (1) presents a tensile stress (*σ_C_t__*) loaded to a concrete section.

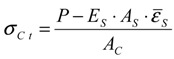
(1)
where *P* is the providing force, *A* is the sectional area, *E* is Young’s modulus and $\bar{\varepsilon }$ is average strain near the mid-span (within 390 mm). Subscripts *C* and *S* stand for concrete and rebar respectively.

### 3.2. Tensile Stress-Strain Response

**Figure 8:**
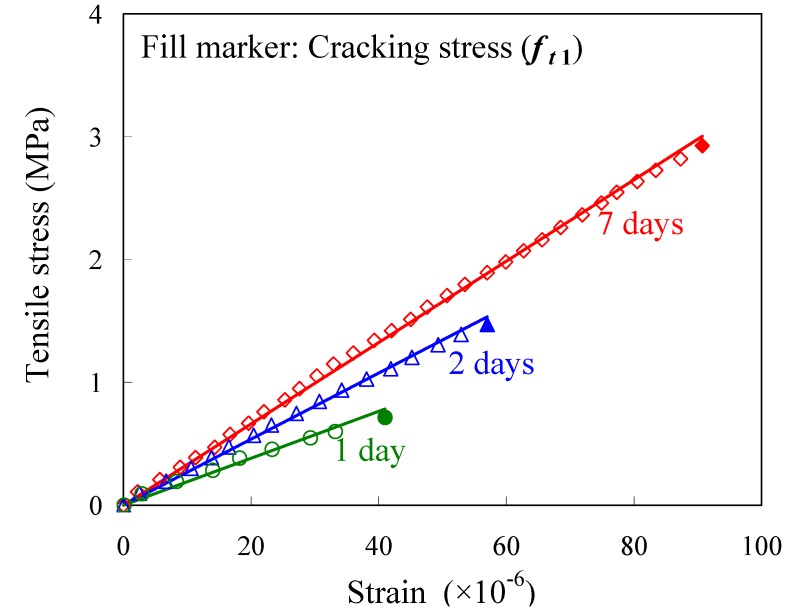
Tensile stress-strain responses (at the ages of 1, 2, 7 days).

Figure 8 shows the tensile stress-strain responses at the ages of 1, 2 and 7 days. Tensile stresses in concrete are estimated by Equation (1), and tensile strains of concrete are obtained from the average strain that is constant in mid-span. All responses obtained in the test were significantly linear until cracking occurred at the maximum strain of each concrete. Uniaxial tensile strength (cracking stress of concrete) can be estimated from the stress at the maximum strain in the responses. Each gradient of the regression line shows tensile Young’s modulus.

### 3.3. Tensile Young’s Modulus

JSCE [20] and ACI-318-08 [21] provide a formula for predicting Young’s modulus of concrete using compressive strength. In addition, EC2-02 (2002) [22] also gives a predicting formula using compressive strength. These formulae are given in Equations (2) and (3), respectively 
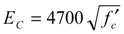
(2)

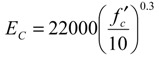
(3) where *E_C_* (MPa) is Young’s modulus of concrete irrespective of compression or tension, and *f’_c_* is the compressive strength (MPa).

Herein, Equation (2) omits the coefficient considering the creep effect from the original formula of JSCE. It should be noted that these formulae are based on the test of compressive Young’s modulus.

**Figure 9:**
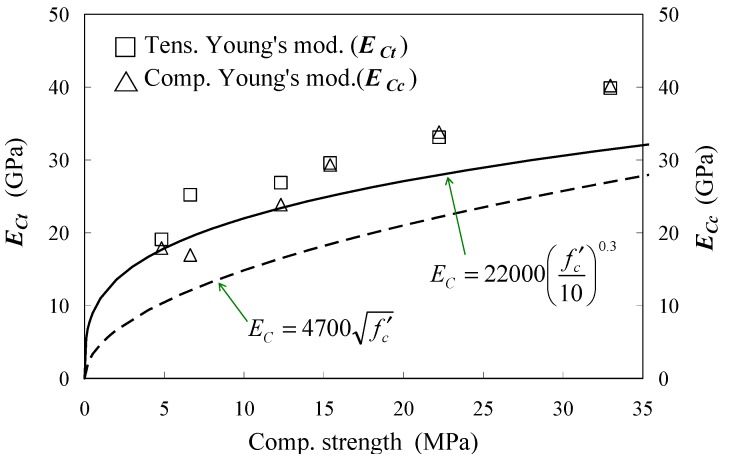
Young’s modulus *vs*. compressive strength.

Figure 9 represents a relation between the tensile Young’s modulus, which is estimated from the gradient of the regression line, and compressive strength at a specific age. For comparison, compressive Young’s moduli obtained from the conventional tests are plotted in the graph. Moreover, the graph includes 2 curves estimated from Equations (2) and (3) as well as the experimental data.

Figure 9 shows that tensile Young’s moduli are greater than compressive moduli, under the compressive strength of approximately 15 MPa. The graph shows both moduli, which are almost equal, over the strength. The strength in this experiment was obtained at an age of 3 days. This result is similar to one found when a direct tension test using dog-bone-shaped concrete specimens was conducted [8]. As shown in Figure 9, estimation curves by Equations (2) and (3) are lower than the experimental results. The comparative result shows that tensile stress may be underestimated when using these formulae. Therefore, employing the tensile Young’s modulus is recommended when estimating tensile stress using the strain of concrete.

### 3.4. Cracking Stress

**Figure 10:**
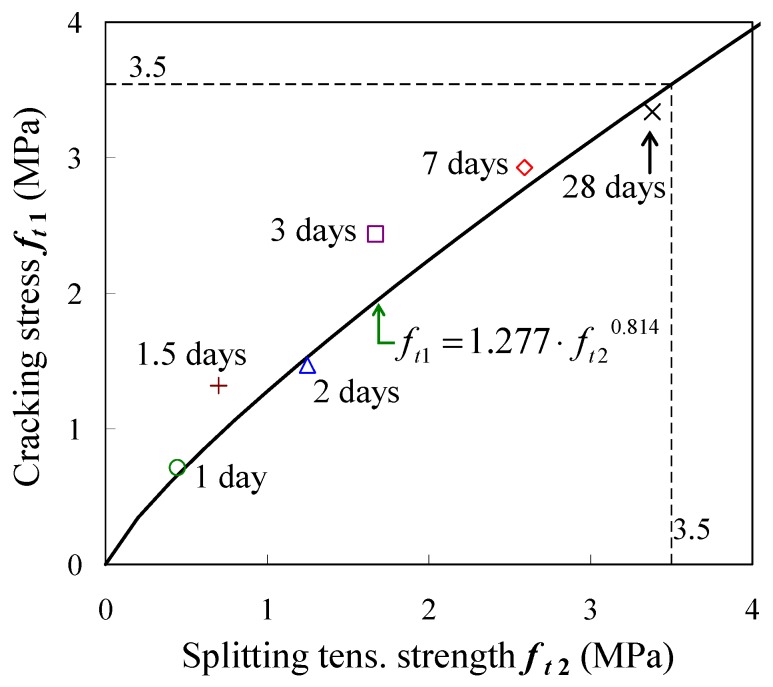
Cracking stress *vs*. splitting tensile strength.

Figure 10 presents the relationship between splitting tensile strength and cracking stress that reaches maximum stress under uniaxial tensile loading. Yoshimoto proposed an empirical equation given in Equation (4) for such a relationship [23]. It should be noted that the object of his study was mature concrete, and the equation was proposed based on a direct tension test using concrete only

*f_t_*_1_ = 1.277 · *f_t_*_2_^0.814^ (4)

where *f_t_*_1_ is the cracking stress (MPa), and *f_t_*_2_ is the splitting tensile strength (MPa).

Cracking stress estimated by the equation becomes almost equal to the splitting tensile strength at 3.5 MPa, and all splitting tensile strengths in the present test are lower than the strength.

Figure 10 demonstrates that the experimental data can be simulated by the estimated curve from the above equation under the strength of 3.5 MPa. The comparative result implies that cracking stress may be estimated using this equation and splitting strength even in early-age concrete.

### 3.5. Tensile Strain Capacity

Table 4 gives the maximum strain of each test, which represents the tensile strain capacity of the concrete. The strain capacity develops according to hydration before the age of 2 days, and becomes constant after the age of 3 days. The constant strain capacity seems to be slightly lower than strain capacities (up to 100–150 × 10^−6^) that were reported in previous investigations [4,24]. The difference may be caused by the restraint strain of concrete shrinkage by the embedded rebar. The restraint of concrete shrinkage induces the tensile pre-stress and the growth of micro cracks around the rebar. Most of concrete structures have rebars near the surface to prevent cracking, so the strain capacity shown in the paper may be considered as conservative data for the design. It should be noted that the mechanical properties presented in this paper such as the tensile Young’s modulus and the cracking stress are also affected by the reinforcing bar.

**Table 4:** Tensile strain capacities and strengths.

**Age (days)**	0.5	1	1.5	2	3	7	28
**Tens. strain capacity (×10^−6^)**	--	41	54	57	86	91	86
**Cracking stress (MPa)**	--	0.71	1.32	1.47	2.44	2.93	3.34
Splitting tensile strength (MPa)*	--	0.44	0.70	1.25	1.67	2.59	3.38
Comp. strength (MPa)*	0.41	4.8	6.6	12.3	15.4	24.6	33.0

*same data shown in Figure 2.

## 4. Bond Properties between Early Age Concrete and Deformed Bars

### 4.1. Evaluation Method for Bond and Slip

**Figure 11:**
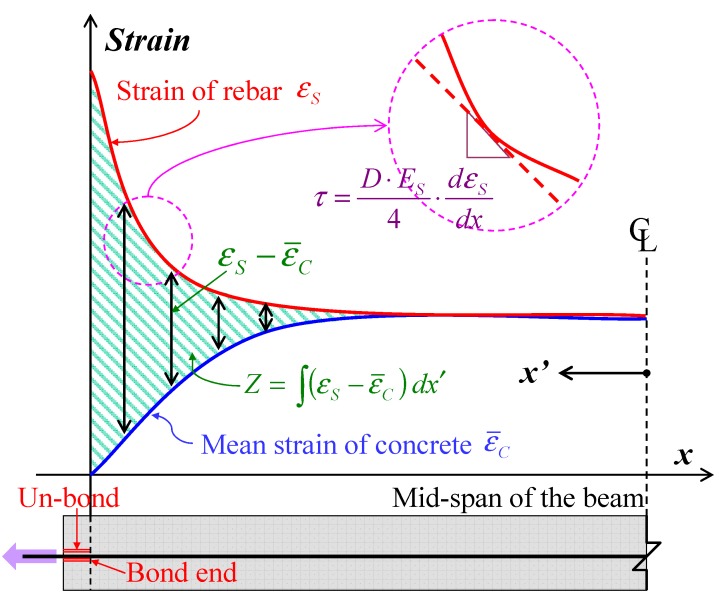
Definitions of slip and bond stress.

In the present study, “slip” is estimated using only the strains of concrete and rebar. The study evaluates the slip by integrating both the strains from the mid-span, which has a boundary condition of zero-slip. That is to say, the sum of the two strains is the slip as given in Equation (5) and Figure 11. 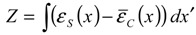
(5) where *Z* is the slip of rebar from concrete and $\bar{\varepsilon }$ means strain; *x* and *x’* are location from the bond end and distance from the mid-span, respectively.

Mimura *et al*. estimated the slip using the equation under the assumption that the strain of concrete is constant in each loading [25]. The assumption is appropriate for the range where the strain of the concrete is equal to the strain of the rebar. However, concrete strain near the bond end is not always equal to the strain of the rebar as illustrated in Figure 11. Thus, this study considers the strain of concrete near the bond end for the estimation of slip. Assuming a concrete element is a linear elastic to tensile stress, the mean strain of concrete can be estimated by using Equation (6). 
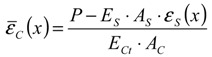
(6)

Mimura *et al*. used a similar equation for estimating the concrete strain, but that study employed compressive Young’s moduli obtained from cylindrical specimens [25]. Tensile Young’s moduli are more appropriate for estimating the tensile strain of concrete. Therefore this study uses the tensile modulus obtained in the previous chapter instead of the compressive modulus.

Equation (7) gives a definition of local bond stress. For simplification, this study assumes the deformed bar as a round bar having a nominal diameter of (13 mm). 
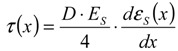
(7)

Yamao *et al*. [26] and Shima *et al*. [27] used two-dimensional curves for estimating the strain distribution, which is a regression curve employing 3 data of strain of rebar. Referring to the method in these investigations, the study estimates the bond stresses. As shown in Equation (7) and Figure 11, the bond stress is estimated by differentiating the strain distribution of rebar.

Herein, JSCE gives a design formula of bond strength (*f_b_*) shown in Equation (8) [20].

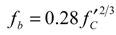
(8)

The formula shows that the bond strength of rebar in concrete is proportional to 2/3 power of compressive strength. To eliminate the influence of the bond strength of various concrete, most research employs standard bond stresses defined as the bond stress divided by 2/3 power of compressive strength. In addition, most research uses the relative slip (*Z/D*). Based on the previous evaluation method, the present study also employs the standard bond stresses and the relative slip. In particular, the method using standard bond stress may be appropriate because the property of the early age concrete varies with time even in the same mix proportion.

### 4.2. Bond Stress-Slip Relation

**Figure 12:**
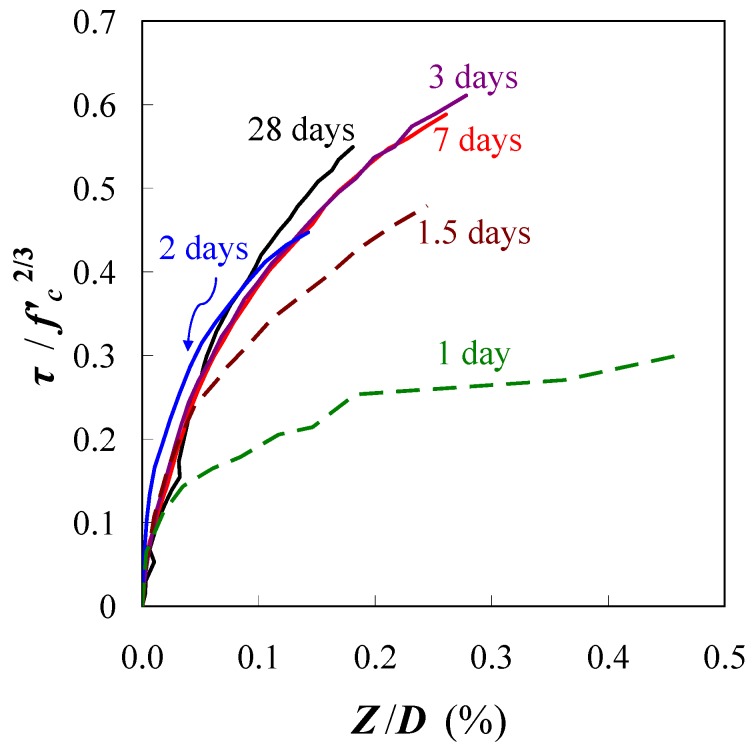
Standard bond stress-relative slip.

Figure 12 shows the relationship between the standard bond stress and the relative slip. The bond stress at the age of 0.5 days was not obtained because the strain of rebar in concrete was completely equal to the strain of un-bonded rebar. Figure 12 shows that the bond stresses at the age of 1 day and 1.5 days are lower than the stress at the other ages. This observation represents the insufficient bond of rebar in the unhardened concrete at a very early age. On the other hand, the bond behavior after the age of 2 days demonstrates almost the same curve as shown in Figure 12. Thus, the bond behavior after this age may be predicted by the relationship for mature concrete. A regression curve for the experiment is given in Equation (9). 
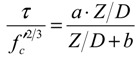
(9) where *a* and *b* are coefficients of the regression curve. The coefficient *a* is 0.39 (1 day), 0.70 (1.5 days) and 0.90 (after 2 days); the coefficient *b* is a constant 0.12.

**Figure 13:**
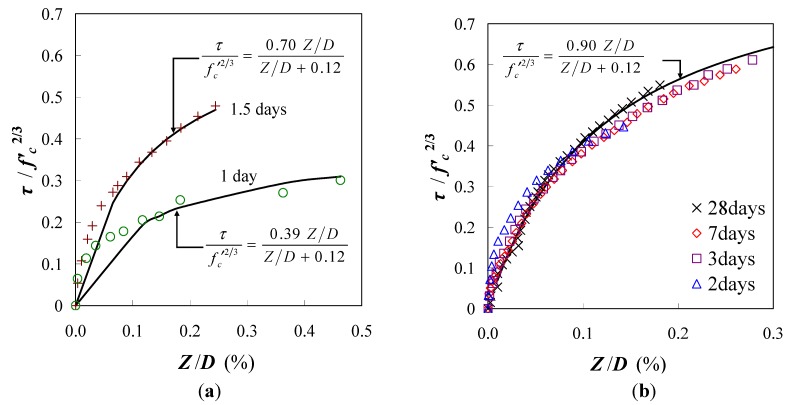
Empirical equations to predict bond behaviors. (**a**) Ages of 1 and 1.5 days; (**b**) Ages of 2, 3, 7 and 28 days.

Figure 13 shows that the data obtained from the experiments can be expressed by using the empirical equation. The coefficient *a* represents the ultimate bond stress; it develops with age and becomes constant after the age of 2 days. Interestingly, the coefficient *b* of all the regression lines in the graph is approximately 0.12. The empirical equations may be useful for predicting the crack width of early age concrete, but they are limited to the mix proportions and materials used in the study.

## 5. Conclusions

The present study aimed to obtain the tensile properties of the concrete which is usually employed in construction of infrastructure in Japan. Based on the uniaxial tension test using RC slender member, this paper describes the cracking stress and bond properties of early age concrete. The conclusions of the study are listed as follows:

(1) Concrete strain can be estimated by using the strain of a rebar having a long embedment length. Tensile stress-strain responses of early age concrete are extremely linear.
(2) Tensile Young’s modulus is higher than compressive modulus of early age concrete, and both moduli gradually become equal as hydration develops over time. The prediction formulae shown in the previous codes may underestimate tensile Young’s modulus of early age concrete.
(3) The empirical equation for mature concrete can be applied to the relationship between uniaxial tensile strength and splitting tensile strength even in early age concrete.
(4) Bond behaviors after the age of 2 days are almost equal, so an equation can be applied to this behavior. The empirical equation based on the test is proposed in the paper.
